# Calls during agonistic interactions vary with arousal and raise audience attention in ravens

**DOI:** 10.1186/s12983-017-0244-7

**Published:** 2017-12-21

**Authors:** Georgine Szipl, Eva Ringler, Michela Spreafico, Thomas Bugnyar

**Affiliations:** 1 Department of Cognitive Biology, University of Vienna, Althanstrasse 14, A-1090 Vienna, Austria; 2 Core Facility Konrad Lorenz Research Station for Behaviour and Cognition, University of Vienna, Fischerau 11, A-4645 Gruenau im Almtal, Austria; 3 Messerli Research Institute, University of Veterinary Medicine Vienna, Medical University of Vienna, and University of Vienna, Veterinaerplatz 1, A-1210 Vienna, Austria; 4 Department of Integrative Zoology, University of Vienna, Althanstrasse 14, A-1090 Vienna, Austria

**Keywords:** Arousal, Common raven, Conflict, Vocal communication, Playback experiment

## Abstract

**Background:**

Acoustic properties of vocalizations can vary with the internal state of the caller, and may serve as reliable indicators for a caller’s emotional state, for example to prevent conflicts. Thus, individuals may associate distinct characteristics in acoustic signals of conspecifics with specific social contexts, and adjust their behaviour accordingly to prevent escalation of conflicts. Common ravens (*Corvus corax*) crowd-forage with individuals of different age classes, sex, and rank, assemble at feeding sites, and engage in agonistic interactions of varying intensity. Attacked individuals frequently utter defensive calls in order to appease the aggressor. Here, we investigated if acoustic properties of defensive calls change with varying levels of aggression, and if bystanders respond to these changes.

**Results:**

Individuals were more likely to utter defensive calls when the attack involved contact aggression, and when the attacker was higher in rank than the victim. Defensive calls produced during intense conflicts were longer and uttered at higher rates, and showed higher fundamental frequency- and amplitude-related measures than calls uttered during low-intensity aggression, indicating arousal-based changes in defensive calls. Playback experiments showed that ravens were more likely to react in response to defensive calls with higher fundamental frequency by orientating towards the speakers as compared to original calls and calls manipulated in duration.

**Conclusions:**

Arousal-based changes are encoded in acoustic parameters of defensive calls in attacked ravens, and bystanders in the audience pay attention to the degree of arousal in attacked conspecifics. Our findings imply that common ravens can regulate conflicts with conspecifics by means of vocalizations, and are able to gather social knowledge from conspecific calls.

**Electronic supplementary material:**

The online version of this article (10.1186/s12983-017-0244-7) contains supplementary material, which is available to authorized users.

## Background

The acoustic structure of vocalizations is modulated by various factors. External stimuli may influence an individuals’ physiological state, which in turn induce changes in the structure of their vocalizations. In social animals, a conspecifics’ behaviour can represent external stimuli that could change the motivational state of a signaller. The emotional state of an individual influences acoustic properties of its vocalizations, with sounds becoming more harsh and lower in frequency when hostility and fear increase [1]. A recent framework proposed a two-dimensional approach to investigate emotional states in animals [2]. Instead of defining basic discrete emotions (e.g. fear, happiness, see [1]) the underlying emotions are measured along two axes. These axes consist of arousal, the physiological activation via the nervous system, and valence, the value of a certain emotion that ranges from very negative to highly positive [2]. Combining the acoustic properties of sounds with their underlying motivation gives insights into the emotional basis of communication during social interactions. The study of different emotional states based on acoustic measures requires a thorough understanding of the mechanisms of sound production and the effects of physiological processes on vocal production [3]. Vocalizations in humans are produced by the vibrating tissue (the source), and then shaped by the vocal tract (the filter) [4]. Although this concept was developed on human speech, it was successfully generalized to mammal vocal production [5, 6] and perception [7–9].

Recent studies suggest that although the vocal apparatus of mammals differs morphologically from the sound-producing organ of birds, the concept of the source-filter theory can still be applied to avian species both from a production [10–18] and a perception side [19, 20]. Hence, source- and filter-related acoustic features known to vary with arousal in mammals (e.g. fundamental and formant frequencies, amplitude, call duration; [21–23]) should cause comparable changes in acoustic parameters also in birds [18]. These acoustic parameters may thus serve as reliable indicators of a caller’s emotional state in general, and may help to manage social interactions with conspecifics and prevent escalation of conflicts. As communication usually occurs in a network of several animals in signalling and receiving range of each other [24], the emotional state of a caller may influence the behaviour of several individuals, addressees in direct interactions and bystanders alike. Consequently, studies should also take into account whether bystanders respond to arousal-based differences in acoustic signals, and thus are capable of inferring the emotional state of the caller.

Common ravens are opportunistic scavengers and gather at large ephemeral food sources such as carcasses [25], where they engage in agonistic interactions of varying intensity with conspecifics [26]. The intensity of an attack can be divided into attacks with and without physical contact: during fights and forced retreats, the aggressor attacks the victim with its beaks and claws, while the victim either fights back, or retreats [27]. During approach-retreat interactions (hereafter ‘retreats’) and submissive displays, the victim is displaced without physical contact. Yet, during submissive displays the aggressor shows self-assertive displays, with erected feathers above the eyes (‘feather-ears’) and the flanks, and the victim signals subordination through a retracted neck and a depressed plumage [27]. Independent of the level of aggression, the victims may utter defensive calls. Ravens were shown to establish a dominance hierarchy that is structured by age, sex, and bonding status: adult birds usually outrank younger ones, males outrank females, and birds with bonding partners outrank singletons [28].

Ravens have a large vocal repertoire [29, 30], including species-typical and individually learned calls. Among the former, many call types are well-studied with respect to call production and function (e.g. food-associated calls: [31–34] and territorial calls: [35, 36]), while comparably little is known about defensive calls. Defensive calls have been described as highly variable in duration, and are uttered as single calls or sequences of several calls when retreating from dominant conspecifics [37, 38]. As only victims call when retreating from aggressors, it seems that defensive calls function to signal distress and subordination, or ‘appeasement’ [39]. The experienced emotions during attacks are almost certainly negative for the victims; however, the level of arousal may vary with the intensity of the aggression and the perceived threat, and therefore should be reflected in the acoustic structure of defensive calls. In mammals, the most prominent changes in vocalizations relate to call duration, call rate, amplitude, and fundamental frequency, with calls becoming longer, higher in rate, louder and harsher with increasing arousal [23].

We here investigated defensive calls of individually marked free-ranging ravens in the Austrian Alps during agonistic encounters of varying intensity in the context of foraging. We first identified agonistic interactions and analyzed whether in addition to the intensity of the attack the opponents’ rank and relatedness influenced calling occurrences. We expected that the propensity to call and the number of calls emitted would vary with the level of aggression, i.e. calling would be more likely and more calls would be uttered when the conflict was more severe. In addition, the propensity to call and the number of calls uttered may vary inversely with fighting ability, whereupon we would expect calling propensity and the number of calls to be higher in low-ranking individuals. Finally, we expected conflicts to occur predominantly between unrelated individuals, as kin were shown to support each other during agonistic interactions [40]. We then analyzed the acoustic structure of defensive calls with special emphasis on acoustic parameters found to relate to arousal in mammals. We expected to find variation in accordance with those shown in mammals [23], e.g. longer and less tonal defensive calls with increasing attack intensity and opponents’ rank disparity.

Defensive calls raise the attention of bystanders [39, 41]. Victims of aggression were shown to receive social support from bystanders that are lower in rank than themselves, that supported them in previous conflicts, and from kin as well as bonding partners [28, 40]. It remains unknown whether calling increases the probability of receiving support, and which acoustic features of defensive calls bystanders pay attention to. Thus, we selected two parameters that showed significant variation in victims’ defensive calls according to the intensity of the attack, and manipulated these parameters experimentally. Using playback experiments, we tested receivers’ abilities to discriminate between natural and manipulated defensive calls. We hypothesized that higher proportions of bystanders would look towards the speaker when playing back calls that simulated increased arousal.

## Methods

### Data collection

Dyadic agonistic interactions were observed ad libitum [42] from August 2010 to July 2012 at the enclosures of wild boars, bears and wolves during morning feedings (0700–0900 a.m.) at the Cumberland Gamepark in Grünau im Almtal, Upper Austria (47°51′ N, 13°57′ O). The gamepark is built into a naturalistic landscape along the river Alm. Free-ranging ravens gather during morning feedings to snatch food from zoo animals, and are well habituated to the presence of human observers at those enclosures.

In the course of an ongoing monitoring project, ravens have been trapped and marked individually using coloured leg rings and metal rings from the German ringing station. The age class (juvenile, subadult, and adult) was determined by the coloration of the inner beak, which is pink in juveniles below 1 year, pinkish with dark speckles in 2 to 3 year olds, and turns completely black in adult birds aged older than 3 years [43]. At the start of the study, 130 ravens had been marked already. Another 74 ravens were marked in the course of the study, totalling 204 marked ravens. As non-breeder ravens are vagrant, the number and identity of birds present at the feedings varied daily and seasonally. An average of 22.97 ± 8.5 (SD) marked ravens were present during daily feedings in the study period (*N* = 516 days). At the onset of each feeding, observers were positioned next to the outer fence of the enclosures and delivered the food to the zoo animals, which prompted the ravens to land inside the enclosure and start foraging. Data was recorded using binoculars and voice recorders. In addition, all foraging ravens were video-taped using a digital camera (Canon HF-11 HD camcorder). From the videos, we coded the identity of both opponents and whether the victim produced defensive calls for each dyadic agonistic interaction between marked individuals. In addition, we coded the occurrence of an intervention by a third party, and whether the third party supported the victim, or the aggressor. From August 2011 to July 2012, sound recordings were conducted in addition to behavioural observations using a Sennheiser ME67 directional microphone (frequency response: 40–20,000 Hz) on a K6 Module connected to a Marantz recording device (Marantz PMD-670). Recordings were conducted at distances of 3–10 m with a sampling rate of 48 kHz and a 16-bit amplitude resolution.

### Dominance hierarchy

Dominance indices were calculated on 942 agonistic interactions using SOCPROG 2.6 with MATLAB R2015a [44]. Modified David’s scores that account for unbalanced interaction rates [45] were extracted of each individual and normalized to obtain scores ranging from 0 to 1. Age class and sex are closely linked to dominance in ravens [28], and also in our data, adult birds outranked subadults and juveniles (Kruskal-Wallis test: H = 23.777, df = 2, *p* < 0.001), and males had a higher rank than females (Mann-Whitney U test: U = 391.0, *p* < 0.001). Thus, only rank differences (rank aggressor - rank receiver) were used in subsequent analyses.

### Factors influencing calling propensity and the number of calls uttered

Generalized Linear Mixed Models (GLMMs) were calculated on 865 agonistic interactions involving 83 marked individuals (468 dyads) using the lme4 package [46] in R [47]. Calling (yes/no) was used as binomial response variable with a logit link function. The full model included the factors level of aggression (fight, forced retreat, retreat, and submission), rank difference of opponents, and kinship of opponents based on DNA analysis (full-sibling/parent-offspring, half-sibling, unrelated; detailed descriptions are provided in the Additional file 1). As random factor the identities of the opponents was entered to account for repeated interactions between opponents. To analyze the number of calls uttered during an agonistic interaction bout, a total of 135 bouts were analyzed with a GLMM using a Poisson distribution and a log link function. The identities of the opponents were used as a random factor. Level of aggression, rank difference of opponents, kinship of opponents, two-way interactions between level of aggression and rank difference and level of aggression and kinship were used as fixed factors. Variance inflation factors were calculated beforehand for all fixed factors in the model to ensure that no collinear parameters were entered in the models [48]. To rank the models, the difference in AICc (ΔAICc) was calculated by subtracting the lowest AICc from all others. As measures of strength of evidence for each model, relative likelihood (exp (−0.5/ΔAICc)) and Akaike weight (relative likelihood/sum of all relative likelihoods) were computed [49]. The models with the highest support were selected based on ΔAICc values (ΔAICc ≦ 2). As several models had high support, models were averaged using the MuMIn package [50] in R [47]. Post hoc pairwise comparisons were conducted using the multcomp package [51] in R [47], which accounted for multiple comparisons. The averaged models are shown in Table 1, the full model selection is presented in the Additional file 1: Table S1.

**Table 1:** Results of averaged models (all models with AICc value ≤2) on the propensity to call, and the number of call per interaction bout, with estimated means (EM), adjusted standard errors (SE), *z* values, and lower and upper confidence intervals (CI)

Coefficients	Level of aggression	EM	Adjusted SE	*z* value	CI	
					2.50%	97.50%
*Calling propensity*						
Intercept		1.871	0.582	3.22	0.73	3.01
Level of aggression	forced retreat^a^	−1.052	0.578	1.82	−2.18	0.08
	retreat^a^	−4.370	0.687	6.36	−5.72	−3.02
	submission^a^	−1.690	0.708	2.39	−3.08	−0.30
	retreat^b^	−3.318	0.344	9.66	−3.99	−2.65
	submission^b^	−0.639	0.409	1.56	−1.44	0.16
	submission^c^	2.680	0.468	5.73	−3.10	−1.90
Rank difference		0.482	0.451	1.07	−0.40	1.37
*Number of calls/bout*						
Intercept		2.143	0.314	6.83	1.53	2.76
Level of aggression	forced retreat^a^	−1.702	0.328	5.19	−2.35	−1.06
	retreat^a^	−1.698	0.452	3.75	−2.58	−0.81
	submission^a^	−0.877	0.404	2.17	−1.67	−0.09
	retreat^b^	0.004	0.339	0.01	−0.66	0.67
	submission^b^	0.825	0.251	3.28	0.33	1.32
	submission^c^	0.821	0.395	2.08	0.05	1.59
Rank difference		−0.159	0.372	0.43	−0.89	0.57

Set as reference:

^a^fight

^b^forced retreat

^c^retreat

### Sound analysis

A total of 377 defensive calls of 30 individuals were analyzed with a custom-built script in Praat [52]. The detailed routine is provided in the Additional file 2. Parameters measured were call duration (s), harmonicity (dB), amplitude measures: mean (dB), minimum (dB), relative time of minimum (%), maximum (dB), relative time of maximum (%), amplitude variation over time (dB/s); measures of the fundamental frequency (*f*o): mean (Hz), minimum (Hz), relative time of minimum (%), maximum (Hz), relative time of maximum (%), range (Hz), start (Hz), end (Hz), and sum of variation (sum of all *f*o changes); jitter; inflex (number of *f*o changes/s); and tonality (relative duration of tonal parts).

To reduce the amount of acoustic variables, a Principle Component Analysis (PCA) was conducted. Call duration loaded on a single component in the PCA (cp. Table S2 in the Additional file 1) and did not group with other acoustic measures, and thus was excluded from the analysis. PCA was recalculated without call duration, and three Principle Components (PCs) with eigenvalues greater than 1.0 were extracted which explained 90.27% of the total variance (Table 2). The Kaiser-Meyer-Olkin measure of sampling adequacy was 0.708, indicating that the data was suitable for PCA. The first extracted PC included *f*o-related variables (mean, minimum, maximum, start and end *f*o) and explained 51.62% of the variance (hereafter termed *f*o component). PC2 was comprised of amplitude-related variables (mean, minimum, maximum) and explained 27.59% of the variance (hereafter termed amplitude component). PC3 grouped the variables tonality and jitter, adding 12.19% to the total variance (hereafter termed jitter and tonality component). Regression scores of the three PCs were extracted. Call duration was analyzed separately using original measured values in seconds instead of regression scores.

**Table 2:** Component matrix with loadings of the PCA

Acoustic Variable	Principal Components		
	1	2	3
Mean *f*o (Hz)	**0.97**	0.18	0.08
Minimum *f*o (Hz)	**0.97**	0.09	−0.01
Maximum *f*o (Hz)	**0.94**	0.20	0.07
End *f*o (Hz)	**0.93**	0.10	−0.01
Start *f*o (Hz)	**0.93**	0.08	−0.02
Mean amplitude (dB)	0.17	**0.97**	0.15
Maximum amplitude (dB)	0.19	**0.96**	0.13
Minimum amplitude (dB)	0.10	**0.96**	0.17
Jitter	−0.16	−0.08	**−0.90**
Tonality	−0.14	0.29	**0.84**
% of variance explained	51.62	27.59	12.19
Eigenvalue	5.16	2.76	1.22

The dimension of the acoustic variables measured from defensive calls (*N* = 377) were reduced to three PCs. Loading higher than 0.5 are highlighted in bold

Linear Mixed Models (LMMs) were calculated for the regression scores of each PC and call duration with a gaussian distribution and an identity link function with the lme4 package [46] in R [47]. As opponents were sampled multiple times, and as victims uttered several calls per interaction bout, a random factor was entered which nested consecutive calls of each interaction bout within the aggressor-victim dyad. The full models included the fixed factors level of aggression, rank difference of opponents, and kinship of opponents. In addition, two-way interactions between level of aggression and rank difference and level of aggression and kinship were added to the full model. All fixed factors in the model were tested for multicollinearity [48]. All models were ranked using relative likelihood and Akaike weights as described above, and the models with the highest support are shown in Table 3. Post hoc pairwise comparisons were done using the multcomp package [51] in R [47] to account for multiple comparisons. The full model selection table is presented in Table S3 in the Additional file 1.

**Table 3:** Model selection table for models with the highest support (Δi≦2) investigating calling occurrences, the number of calls per interaction bout, the three PCs, call duration, and the responses to playbacks of defensive calls manipulated in *f*o and call duration

Response	Model No.	Fixed factors	AICc	Δi	Relative likelihood	Akaike weight
*Calling propensity*	1	Level of aggression	960.4	0.0	1.0	0.50
	2	Level of aggression + rank difference	961.2	0.9	0.65	0.33
*Number of calls/bout*	1	Level of aggression	528.0	0.0	1.0	0.49
	2	Level of aggression + rank difference	530.1	2.0	0.36	0.18
*fo component*	1	Level of aggression * rank difference	823.1	0.0	1.0	0.78
*Amplitude component*	1	Level of aggression	635.6	0.0	1.0	0.58
*Jitter and tonality component*	1	Rank difference	987.5	0.0	1.0	0.72
*Call duration*	1	Level of aggression * rank difference	−1310.4	0.0	1.0	0.87
			QAICc	Δi	Relative likelihood	QAkaike weight
*Pitch manipulation*	1	Manipulation type	37.1	0.0	1.0	0.61
*Duration manipulation*	1	Null	60.2	0.0	1.0	0.72

Corrected Akaike Information Criterion (AICc) values, the difference between the lowest AICc value and all other AICc values (Δi), the relative likelihood, and resulting Akaike weights are presented (for the models on the playback experiment, quasi (Q) values are shown). “*” indicate main factors and their two-way interaction

Individual discrimination was tested with a permutated discriminant function analysis (pDFA; [53]) in R [47]. A crossed pDFA with 1000 permutations was calculated on a fully balanced set of 115 calls of 23 individuals (5 calls per individual) using the three PC scores and call duration.

### Playback experiment and analysis

Eight defensive calls of four male and four female adult ravens with known identity were selected with little background noise and no overlapping calls of other birds. All calls were similar in duration (mean ± SD: 0.187 s ± 0.024) and mean *f*o (mean ± SD: 447.89 Hz ± 17.33). Calls were adjusted to the lowest sound pressure level using Sound Booth for Mac to assure that all calls had the same sound pressure level. Duration and *f*o manipulations were conducted in Praat [52]. Each call was shortened and lengthened by 50%, and *f*o was shifted up and down by 100 Hz. The routine used in Praat is described in the Additional file 1. We designed a playback experiment to test responses, defined as head turns towards the speaker, of free-ranging ravens to defensive calls manipulated in frequency and duration. We conducted 8 sessions to test responses to duration manipulations, and 8 sessions to test responses to frequency manipulations. In each session, we played three calls, the original, unmanipulated defensive call, and two calls either manipulated in duration (shorter and longer) or in *f*o (shifted up and down by 100 Hz) in randomized order. Sessions testing duration and *f*o were alternated. The minimum interval between two played back calls in a session was 2 min, and the minimum interval between two sessions was 1 week. Playbacks were conducted during morning feedings. Thirty minutes prior to the feeding, a battery-powered loudspeaker (Roadboy 65, LD Systems, frequency response: 80–15,000 Hz) was placed approximately 3 m from the fence of the wild boar enclosure, and concealed with a camouflage net. When feeding started, the food was provided to the wild boars, causing the ravens to descend and to start scrounging food. Playback stimuli were presented approximately 10 min after the start of the feeding using an iPod nano (6th generation, http://www.apple.com). The iPod was connected to the speaker via a radio transmitter-receiver system (Sennheiser EW 112-p G3-A Band, 516e558 MHz), allowing the playback to be conducted without a visible connection of the experimenter to the speaker. Each session was videotaped using a HD digital camera (Canon HF-11 HD camcorder) on a tripod, which allowed us to precisely measure the responses. The number of birds present and the number of birds responding by turning their head towards the speaker was scored from the videos. Additionally, we scored the number of defensive calls that were uttered within 1 min prior to the playbacks.

Responses of ravens to the playbacks of defensive calls were analyzed with a logistic regression model in R [47]. Separate models were calculated for sessions testing responses to duration and session testing responses to *f*o using a quasibinomial distribution and a logit link function to account for overdispersion. As response variable, we used a vector that was created from the number of responding birds (successes) and the number of birds that did not respond (failures) to account for varying numbers of birds present in different sessions (mean number of birds present ± SD = 8.66 ± 4.91 individuals). Manipulation type (original, *f*o shifted up, *f*o shifted down or original, shorter, longer), the sex of the bird used as stimulus, and the number of defensive calls per minute prior to the playback were used as fixed factors in the full model. To rank the models, quasiAICc values (QAICc) were calculated by dividing the residual deviance (−2 log-likelihood) with the overdispersion parameter of the full model [54]. From this, ΔQAICc, relative likelihood (exp (−0.5/ΔQAICc)) and quasi Akaike weights were computed (Table S4 in the Additional file 1).

## Results

### Patterns of agonistic interactions

Out of 865 observed agonistic interactions between marked individuals, the majority were initiated by adult ravens and directed towards other adults or subadults. While males were targeting both sexes, females tended to focus on other females (see Table S5 in the Additional file 1). Subadult birds showed a similar pattern, but initiated less conflicts, and juveniles hardly initiated agonistic interactions at all. In 68.9% of all agonistic interactions the opponents were unrelated, 24.3% occurred between half-siblings, and only 6.8% of the dyads were between full-siblings. Interventions in agonistic interactions between individually marked individuals were observed 63 times; in 44 instances the third party supported the aggressor, targeting the victim, and the victim received support in 19 cases.

### Factors influencing calling propensity and the number of calls uttered

**Fig. 1:**
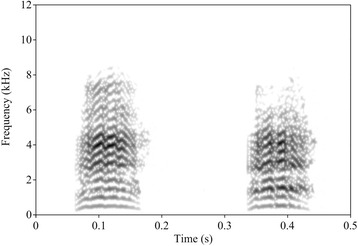
Example of two defensive calls. Spectrogram settings: FFT method, Gaussian window shape, window length = 0.01 s, time steps = 700, frequency steps = 250, dynamic range = 70 dB

Ravens uttered defensive calls in 51.9% of all agonistic interactions (cp. Table S5). Victims tended to receive support from a third party more often when calling (14 out of 19 cases with calling: Chi-squared (1) = 3.37, *p*-value = 0.067). Defensive calls had an average duration of 0.140 ± 0.05 s (SD) and were strongly time-frequency modulated (for an example of two defensive calls see Fig. 1, for descriptive measures see Table S6 in the Additional file 1).

**Fig. 2:**
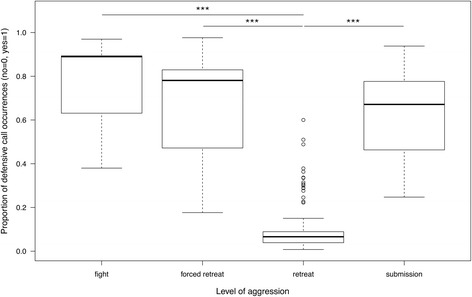
Estimated mean proportion of defensive call occurrences for different levels of aggression. Whiskers represent 1.5XIQR, bold lines denote the median, and circles show outliers. Asterisk indicate p≦0.001

The level of aggression (reflecting the intensity of a conflict) affected the birds’ propensity to call. The victims produced defensive calls in 75.8% of all fights and 65.4% of all forced retreats. Submissive displays were accompanied by defensive calls in 60.0% of the cases, and (low-intensity) approach-retreat interactions triggered calls in only 12.6% of the cases. The averaged model identified the level of aggression as the most important factor (relative importance: 1.0), and the rank difference of opponents as the second factor (relative importance: 0.39; Table 1). This indicates that dominance relationships were, aside from the level of aggression, a key factor to understand why victims produced defensive calls. Pairwise comparisons on the averaged model showed that the proportion of calling was significantly lower during approach-retreat interactions as compared to fights, forced retreats, and submissive displays (Fig. 2).

**Fig. 3:**
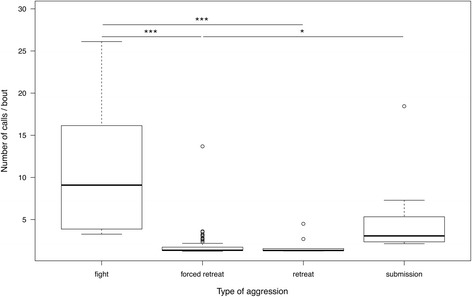
Estimated mean number of defensive calls per interaction bout for different levels of aggression. Whiskers represent 1.5XIQR, bold lines show the median, and circles indicate outliers. Asterisk indicate *p*≦0.001 (***) and *p*≦0.05 (*)

The number of calls per interaction bout was also influenced by the level of aggression (relative importance: 1.0) and the rank difference of opponents (relative importance: 0.27; Table 1). The highest number of calls per interaction bout was found during fights (Fig. 3). Fewer calls were uttered during submissive displays, and the lowest number of calls were found for forced retreats and approach-retreat interactions. Victims uttered higher numbers of calls per interactions bout when opponents had higher rank differences; i.e. when the victims were very low-ranking, and the aggressors were high-ranking individuals.

### Acoustic structure

**Fig. 4:**
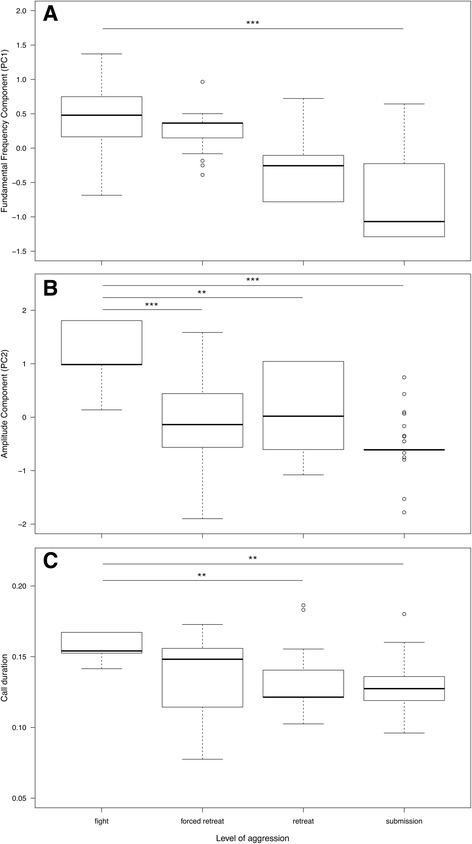
Estimated mean values for the *f*o component (**a**), the amplitude component (**b**), and call duration (C) with regard to the level of aggression that elicited the defensive calls. Values were derived from the model with the highest support (Table 4). Bold lines indicate the median. Whiskers show 1.5XIQR, and circles denote outliers. Asterisk show adjusted *p* values corrected for repeated testing and indicate *p*≦0.001 (***) and *p*≦0.01 (**)

The level of aggression had a strong effect on the *f*o component (F = 4.59, df = 3, *p* = 0.004), the amplitude component (F = 6.12, df = 3, *p* < 0.001), and call duration (F = 3.51, df = 3, *p* = 0.027; Table 3). In all these parameters, highest values were found for defensive call uttered during fights (Fig. 4, Table 4). This supports our hypothesis that these acoustic parameters indicate arousal-based changes in defensive calls. Values decreased gradually for forced retreats and retreats and were lowest during submissive displays.

**Table 4:** Results of the models with the highest support investigating the three PCs and call duration, showing estimated means (EM), adjusted standard errors (SE), *t* values, and lower and upper confidence intervals (CI) of all coefficients

Coefficients	Level of aggression	EM	SE	*t* value	CI	
					2.50%	97.50%
*fo component*						
Intercept		0.098	0.299	0.33	−0.49	0.69
Level of aggression	forced retreat^a^	0.521	0.318	1.64	−0.11	1.15
	retreat^a^	0.303	0.471	0.64	−0.63	1.23
	submission^a^	−0.659	0.375	−1.76	−1.40	0.10
	retreat^b^	−0.218	0.382	−0.57	−0.97	0.54
	submission^b^	−1.180	0.249	−4.74	−1.68	−0.68
	submission^c^	−0.962	0.430	−2.24	−1.81	−0.10
Rank difference		−0.791	2.084	−0.38	−4.94	3.35
Level of aggression: rank difference	forced retreat^a^: rank	0.663	2.114	0.31	−3.54	4.86
	retreat^a^: rank	0.155	2.361	0.07	−4.53	4.83
	submission^a^: rank	2.635	2.138	1.23	−1.61	6.89
	retreat^b^: rank	−0.508	1.154	−0.44	−2.79	1.77
	submission^b^: rank	1.973	0.554	3.56	0.86	3.09
	submission^c^: rank	2.481	1.199	2.07	0.09	4.87
*Amplitude component*						
Intercept		0.820	0.273	3.00	0.27	1.36
Level of aggression	forced retreat^a^	−1.029	0.276	−3.73	−1.57	−0.47
	retreat^a^	−1.250	0.361	−3.46	−1.96	−0.52
	submission^a^	−1.354	0.325	−4.17	−2.00	−0.71
	retreat^b^	−0.221	0.245	−0.90	−0.71	0.27
	submission^b^	−0.325	0.188	−1.73	−0.71	0.05
	submission^c^	−0.104	0.287	−0.36	−0.69	0.47
*Jitter and tonality component*						
Intercept		0.345	0.118	2.93	0.11	0.58
Rank difference		−1.043	0.337	−3.09	−1.72	−0.37
*Call duration*						
Intercept		0.155	0.013	1.19	0.13	0.18
Level of aggression	forced retreat^a^	−0.019	0.014	−1.33	−0.05	0.01
	retreat^a^	0.046	0.022	2.09	0.00	0.09
	submission^a^	−0.029	0.017	−1.74	−0.06	0.00
	retreat^b^	0.065	0.019	3.47	0.03	0.10
	submission^b^	−0.011	0.012	−0.89	−0.03	0.01
	submission^c^	−0.075	0.021	−3.63	−0.12	−0.03
Rank difference		0.057	0.085	0.67	−0.11	0.23
Level of aggression: rank difference	forced retreat^a^: rank	−0.080	0.087	−0.92	−0.26	0.09
	retreat^a^: rank	−0.300	0.103	−2.91	−0.51	−0.10
	submission^a^: rank	−0.005	0.088	−0.06	−0.19	0.17
	retreat^b^: rank	−0.220	0.060	−3.69	−0.34	−0.10
	submission^b^: rank	0.075	0.026	2.87	0.02	0.13
	submission^c^: rank	0.295	0.061	4.82	0.17	0.42

Set as reference:

^a^fight

^b^forced retreat

^c^retreat

“:” indicate interactions between factors

In addition, rank difference of opponents affected variation in the *f*o component (F = 2.17, df = 1, *p* = 0.14), the tonality and jitter component (F = 9.57, df = 1, *p* = 0.002), and call duration (F = 0.13, df = 1, *p* = 0.15). While no clear pattern could be observed for rank difference and the *f*o component and call duration, the tonality and jitter component showed a negative relationship with rank difference: scores decreased, i.e. calls became harsher as rank disparity increased.

The model with the highest support to explain variation in the *f*o component and call duration further included the two-way interaction between the level of aggression and rank difference (*f*o component: F = 4.68, df = 3, *p* = 0.004; call duration: F = 8.79, df = 3, *p* < 0.001; cp Table 3). Both the *f*o component and call duration showed the same pattern: *f*o scores increased and calls became longer as rank disparity increased for submissive displays.

The pDFA failed to discriminate individuals based on the acoustic structure of their defensive calls. None of the cross-validated calls could be classified correctly.

### Playback experiment

**Fig. 5:**
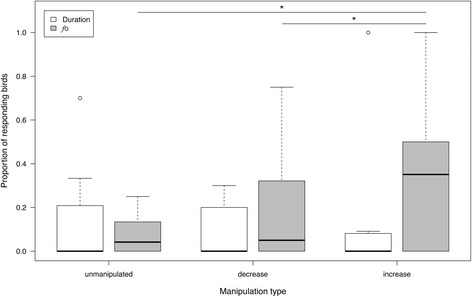
Proportion of responding birds with respect to natural playback stimuli (unmanipulated) and stimuli manipulated in call duration (white boxes) and *f*o (grey boxes). Bold lines indicate the median, and circles the outliers. Whiskers represent 1.5XIQR and asterisk indicate *p* < 0.05 (*)

When playing back calls that varied in *f*o, the model with the highest support included the factor manipulation type (F = 4.44, df = 2, *p* = 0.025; Table 3). Higher proportions of responses were found when *f*o was increased compared to unmanipulated calls and calls with lower *f*o; the latter two treatments showed no difference in the proportions of responses (Fig. 5). When testing differences in proportions of responses to the manipulation of call duration, neither of the factors remained in the model with the highest support, and responses did not differ between the original and the manipulated calls (see Fig. 5).

## Discussion

We here show for the first time that the arousal-based variation in acoustic features previously found in mammals [23] can be found also in birds, and that bystanders are attentive to these experimentally induced changes in conspecific defensive calls. Ravens’ defensive calls showed higher measured of acoustic parameters related to *f*o and amplitude during more intense conflicts. Moreover, bystander ravens were highly attentive to defensive calls with increased *f*o in the playback experiment.

### Calling propensity and number of calls uttered

Victims were most likely to utter defensive calls during intense conflicts with contact aggression such as fights and forced retreats, and during submissive displays that are accompanied by self-assertive displays of the aggressors, and reflect a harassment of the victim. Likewise, the number of calls uttered by the victims were highest during fights and submissive displays. Our findings thus support the hypothesis that high arousal during conflicts may have induced higher calling rates in victims. Similar links were shown between the propensity of calling and call rates and increased arousal in the context of predation for rhesus macaques (*Macaca mulatta*): individuals were less likely to produce alarm calls when treated with an inhibitor of glucocorticoid, and did so at lower rates [55]. Non-invasive studies showed similar results for yellow-bellied marmots (*Marmota flaviventris*), where individuals with higher glucocorticoid levels, and thus higher levels of physiological arousal, were more likely to emit alarm calls in dangerous situations [56]. A recent study revealed that common marmosets (*Callithrix jacchus*) were more likely to produce contact calls with higher arousal, which was measured by heart rate [57].

Defensive calling in ravens was also more likely whenever the rank difference of opponents was high. In ravens, dominance rank is strongly influenced by sex and age class [28, 58]. Thus, a large disparity between victims’ and aggressors’ rank may induce higher arousal, and result in higher calling propensity and higher call rates. Previous findings from captive ravens showed that kin had more valuable relationships than unrelated individuals [59]. Our analysis also showed that genetically related individuals rarely engaged in aggressive interactions with each other, and the factor kinship was not included in the models with the highest support.

### Acoustic structure

The *f*o component increased during agonistic interactions with physical aggression, which indicates that arousal could have influenced the increase of *f*o measures as well. The same was found for the amplitude component, which combined amplitude-related measures of ravens’ defensive calls. These results are in line with previous studies reporting an arousal-based increase in *f*o and relative amplitude in mammals (reviewed in [23]), and in a bird [60]. Likewise, with increased arousal, call duration was reported to increase in some mammals [23], which was also the case for ravens’ defensive calls during high intensity aggression (e.g. fights). However, not only arousal, but also valence may impact on acoustic parameters of vocalizations [2]. Studies investigating valence in avian vocalizations are scarce, and results of studies in mammals are inconclusive, probably because valence is difficult to assess in non-human animals in general [23]. Yet, the duration and rates of vocalizations were shown to be shorter in positive situations [23]. The jitter and tonality component did not vary with the level of aggression. According to the motivational-structural rule, an increase in arousal is expected to influence tonality, or harmonic-to-noise ratio, with sounds becoming harsher, i.e. lower in tonality [1]. On the contrary, some vocalizations were reported to be less noisy or harsh with increased arousal in mammals [23]. The jitter and tonality component was, however, linked negatively with rank difference of opponents, as scores decreased as rank disparity increased. Calls thus became more harsh when the aggressor was very high-ranking and the victim very low in rank, indicating that a high rank disparity may induce a higher threat, and thus higher arousal.

During submissive displays, the intensity of the agonistic interaction and rank disparity shaped acoustic parameters at the same time: the *f*o component scores and call duration increased with rank disparity, indicating that submissive displays are perceived as highly arousing, possibly due to the simultaneous self-assertive displays of the high-ranking aggressors. Thus, defensive calls uttered during submission may signal subordination in order to appease the opponent and to prevent an escalation of the situation.

Defensive calls did not differ between individuals. A possible reason could be that victims may not need to communicate their identity because defensive calls are directed at the aggressor, and the aggressor already knows the identity of the victim prior to the attack. We suggest further studies to investigate whether ravens are effectively not able to recognize or discriminate individuals by their defensive calls.

### Playback experiment

When investigating ravens’ responses to arousal-based changes in defensive calls, the proportion of responding birds, corrected for the total number of birds present, was highest for defensive calls with higher *f*o. This indicates that bystander ravens are attentive to the degree of arousal in attacked conspecifics. However, we did not find increased responses to defensive calls manipulated in duration. It is likely that the presentation of a single call did not elicit strong responses. Victims often uttered several defensive calls in a row during intense conflicts. As ravens only responded to call with increased *f*o, a possible conclusion is that single calls with moderate *f*o of any length do not raise the attention of bystanders because they do not sound highly urgent and aroused, and arousal is encoded in a high rate of calls with increased *f*o. This remains to be tested in future studies that explore responses to changes of other acoustic parameters independently [61, 62] or simultaneously. Another possible reason for low numbers of responding birds could have been the absence of visual cues (e.g. an ongoing conflict).

## Conclusion

Our results show that agonistic interactions that induced high arousal and negative valence influenced the victims’ likelihood to call and the number of calls produced. Furthermore, the acoustic properties of defensive calls were affected by the intensity of the conflicts that induced calling. Variation in acoustic parameters related to *f*o, amplitude, call rate and duration approximate the most commonly varying source-related parameters in the study of vocal communication of emotions in mammals (reviewed in [23]). Our study shows that the same acoustic cues connote negative emotions also in ravens. Furthermore, we show that ravens are attentive to changes in acoustic properties of victims’ defensive calls. This finding implies that bystanders are sensitive to the degree of arousal in attacked birds, and that defensive calls may serve to regulate agonistic social interactions with conspecifics. Corvids’ social and cognitive skills are in many aspects comparable to those found in other highly social species. Their social organization characterized by high fission-fusion dynamics requires that members of subgroups constantly refresh their knowledge of others’ social relationships, which could have changes during prolonged fission periods [63]. One possibility to regain knowledge quickly is through eavesdropping on a variety of social signals. Our findings thus add to our understanding of the communicative value of acoustic signals.

## Additional files

Additional file 1: Detailed methods on kinship analysis and sound preparation for playback experiments, and Tables S1-S6. (PDF 188 kb)

Additional file 2: Praat script used to analyze defensive calls in common ravens. (TXT 4.30 kb)

## References

[1] Morton ES (1977). On the occurrence and significance of motivation-structural rules in some bird and mammal sounds. Am Nat.

[2] Mendl M, Burman OHP, Paul ES (2010). An integrative and functional framework for the study of animal emotion and mood. Proc R Soc Lond B Biol Sci.

[3] Taylor AM, Reby D (2009). The contribution of source-filter theory to mammal vocal communication research. J Zool.

[4] Fant G. Acoustic theory of speech production. The Hague; 1960.

[5] Fitch WT, Hauser MD (1995). Vocal production in nonhuman primates: acoustics, physiology, and functional constraints on “honest” advertisement. Am J Primatol.

[6] Owren MJ, Rendall D (2001). Sound on the rebound: bringing form and function back to the forefront in understanding nonhuman primate vocal signaling. Evol Anthropol.

[7] Fitch WT, Fritz JB (2006). Rhesus macaques spontaneously perceive formants in conspecific vocalizations. J Acoust Soc Am.

[8] Fitch WT (1997). Vocal tract length and formant frequency dispersion correlate with body size in rhesus macaques. J Acoust Soc Am.

[9] Charlton BD, Ellis WAH, Larkin R, Fitch WT. Perception of size-related formant information in male koalas (*Phascolarctos cinereus*). Anim Cogn. 2012;15:999–1006. 10.1007/s10071-012-0527-5 22740017

[10] Beckers GJL, Suthers RA, ten Cate C (2003). Pure-tone birdsong by resonance filtering of harmonic overtones. PNAS.

[11] Beckers GJL, Nelson BS, Suthers RA (2004). Vocal-tract filtering by lingual articulation in a parrot. Curr Biol.

[12] Ohms VR, Snelderwaard PC, ten Cate C, Beckers GJL (2010). Vocal tract articulation in zebra finches. PLoS One.

[13] Nowicki S (1987). Vocal tract resonances in oscine bird sound production: evidence from birdsongs in a helium atmosphere. Nature.

[14] Hoese WJ, Podos J, Boetticher NC, Nowicki S (2000). Vocal tract function in birdsong production: experimental manipulation of beak movements. J Exp Biol.

[15] Riede T, Suthers RA, Fletcher NH, Blevins WE (2006). Songbirds tune their vocal tract to the fundamental frequency of their song. PNAS.

[16] Patterson DK (1994). A comparative study of human and parrot phonation: acoustic and articulatory correlates of vowels. J Acoust Soc Am.

[17] Elemans CPH (2014). The singer and the song: the neuromechanics of avian sound production. Curr Opin Neurobiol.

[18] Elemans CPH, Rasmussen JH, Herbst CT, Düring DN, Zollinger SA, Brumm H (2015). Universal mechanisms of sound production and control in birds and mammals. Nat Commun.

[19] Fitch WT, Kelley JP. Perception of vocal tract resonances by whooping Cranes *Grus americana*. Ethology. 2000;106:559–74.

[20] Dooling RJ, Best CT, Brown SD (1995). Discrimination of synthetic full-formant and sinewave /ra–la/ continua by budgerigars (*Melopsittacus undulatus*) and zebra finches (*Taeniopygia guttata*). J Acoust Soc Am.

[21] Scherer KR (1986). Vocal affect expression: a review and a model for future research. Psychol Bull.

[22] Scherer KR (2003). Vocal communication of emotion: a review of research paradigms. Speech Commun.

[23] Briefer EF (2012). Vocal expression of emotions in mammals: mechanisms of production and evidence. J Zool.

[24] McGregor PK. In: PK MG, editor. Animal communication networks: Cambridge University Press; 2005.

[25] Ratcliffe D (1997). The raven.

[26] Braun A, Walsdorff T, Fraser ON, Bugnyar T (2012). Socialized sub-groups in a temporary stable raven flock?. J Ornithol.

[27] Goodwin D. Crows of the world. 1st ed. London: British Museum (Natural History); 1976.

[28] Braun A, Bugnyar T (2012). Social bonds and rank acquisition in raven nonbreeder aggregations. Anim Behav.

[29] Heinrich B (1989). Ravens in winter.

[30] Enggist-Dueblin P, Pfister U (2002). Cultural transmission of vocalizations in ravens, *Corvus corax*. Anim Behav.

[31] Bugnyar T, Kijne M, Kotrschal K (2001). Food calling in ravens: are yells referential signals?. Anim Behav.

[32] Szipl G, Boeckle M, Wascher CAF, Spreafico M, Bugnyar T (2015). With whom to dine? Ravens' responses to food-associated calls depend on individual characteristics of the caller. Anim Behav.

[33] Boeckle M, Szipl G, Bugnyar T (2012). Who wants food? Individual characteristics in raven yells. Anim Behav.

[34] Heinrich B, Marzluff JM (1991). Do common ravens yell because they want to attract others?. Behav Ecol Sociobiol.

[35] Boeckle M, Bugnyar T (2012). Long-term memory for affiliates in ravens. Curr Biol.

[36] Reber SA, Boeckle M, Szipl G, Janisch J, Bugnyar T, Fitch WT (2016). Territorial raven pairs are sensitive to structural changes in simulated acoustic displays of conspecifics. Anim Behav.

[37] Pfister U (1988). Zur Morphologie, Ontogenese und Funktion der Rufe von Kolkraben.

[38] Gwinner E (1964). Untersuchungen über das Ausdrucks- und Sozialverhalten des Kolkraben (*Corvus corax corax* L.). Z Tierpsychol.

[39] Heinrich B, Marzluff JM, Marzluff CS (1993). Common ravens are attracted by appeasement calls of food discoverers when attacked. Auk.

[40] Fraser ON, Bugnyar T (2012). Reciprocity of agonistic support in ravens. Anim Behav.

[41] Massen JJM, Pašukonis A, Schmidt J, Bugnyar T (2014). Ravens notice dominance reversals among conspecifics within and outside their social group. Nat Commun.

[42] Altmann J (1974). Observational study of behavior: sampling methods. Behaviour.

[43] Heinrich B, Marzluff JM (1992). Age and mouth color in common ravens. Condor.

[44] Whitehead H (2009). SOCPROG Programs: analysing animal social structures. Behav Ecol Sociobiol.

[45] de Vries H, Stevens JMG, Vervaecke H (2006). Measuring and testing the steepness of dominance hierarchies. Anim Behav.

[46] Bates D, Maechler M, Bolker B, Walker S (2015). Fitting linear mixed-effects models using lme4. J Stat Softw..

[47] R Core Team. R: A language and environment for statistical computing. 3rd ed. Vienna: R Foundation for Statistical Computing; 2017. https://www.r-project.org.

[48] Zuur A, Ieno EN, Walker N, Saveliev AA, Smith GM, Gail M, Krickeberg K, Samet JM, Tsiatis A, Wong W (2009). Mixed effects models and extensions in ecology with R. Statistics for biology and health.

[49] Burnham KP, Anderson DR, Huyvaert KP (2011). AIC model selection and multimodel inference in behavioral ecology: some background, observations, and comparisons. Behav Ecol Sociobiol.

[50] Bartoń K (2009). MuMIn: multi-model inference. R package. R package.

[51] Hothorn T, Bretz F, Westfall P (2008). Simultaneous inference in general parametric models. Biom J.

[52] Boersma P, Weenink D. Praat: doing phonetics by computer. 5 ed. 2016. Available from: http://www.fon.hum.uva.nl/praat/. Retrieved 24 May 2015.

[53] Mundry R, Sommer C (2007). Discriminant function analysis with nonindependent data: consequences and an alternative. Anim Behav.

[54] Burnham KP, Anderson DR. Model selection and multimodel inference: Springer; 2012.

[55] Bercovitch FB, Hauser MD, Jones JH (1995). The endocrine stress response and alarm vocalizations in rhesus macaques. Anim Behav.

[56] Blumstein DT, Patton ML, Saltzman W (2006). Faecal glucocorticoid metabolites and alarm calling in free-living yellow-bellied marmots. Biol Lett.

[57] Borjon JI, Takahashi DY, Cordero Cervantes D, Ghazanfar AA. Arousal dynamics drive vocal production in marmoset monkeys. J Neurophysiol. 2016:116;753–64. 10.1152/jn.00136.2016 PMC6208312 27250909

[58] Heinrich B (1994). Dominance and weight changes in the common raven *Corvus corax*. Anim Behav.

[59] Fraser ON, Bugnyar T (2010). The quality of social relationships in ravens. Anim Behav.

[60] Szipl G, Boeckle M, Werner SAB, Kotrschal K. Mate recognition and expression of affective state in Croop calls of northern bald ibis (*Geronticus eremita*). PLoS ONE. Public Library of Science; 2014;9:e88265. 10.1371/journal.pone.0088265 PMC3914947 24505455

[61] Pitcher BJ, Briefer EF, McElligott AG. Intrasexual selection drives sensitivity to pitch, formants and duration in the competitive calls of fallow bucks. Evol Biol. 2015;15:1–13. 10.1186/s12862-015-0429-7 PMC4538740 26279584

[62] Charlton BD, Zhihe Z, Snyder RJ (2010). Giant pandas perceive and attend to formant frequency variation in male bleats. Anim Behav.

[63] Aureli F, Schaffner CM, Boesch C, Bearder SK, Call J, Chapman CA (2008). Fission-fusion dynamics: new research frameworks. Curr Anthropol.

[64] ABS A. Guidelines for the treatment of animals in behavioural research and teaching. Anim Behav. 2016;111:I–IX. 10.1006/anbe.1999.1349 10640387

