# Supplementary material for: Calls during agonistic interactions vary with arousal and raise audience attention in ravens
Source: Front Zool. 2017 Dec 21;14:57. doi: 10.1186/s12983-017-0244-7 (PMC5740903; doi:10.1186/s12983-017-0244-7)
Supplement: Supplementary file 1 — Detailed methods on kinship analysis and sound preparation for playback experiments, and Tables S1-S6. (PDF 188 kb) [file 12983_2017_244_MOESM1_ESM.pdf]

Supplemental Materials

Kinship analysis

Genomic DNA was isolated from blood samples of free-ranging Common ravens using a Proteinase K digestion, followed by a standard phenol–chloroform protocol [1]. All individuals were genotyped at 15 microsatellite loci to obtain individual genotypes for relatedness analyses. PCR amplifications were performed using reaction volumes of 10µL, which contained 20-50ng of genomic DNA, 0.2mm of dNTP, 1µm of forward and reverse primer, 0.5U of Taq DNA polymerase (Axon), and 1µL of 10× NH_4 reaction buffer (Axon) at a final concentration of 1.5 mm $MgCl_2$. The PCR programme applied first performed a denaturation with an initial cycle at 95 °C for 8 minutes, followed by 39 cycles at 95 °C for 45 seconds. Then the annealing phase with the primer-specific annealing temperature was run for 45 seconds. As a final step the extension was performed at 72 °C for 45 seconds, followed by a last cycle for 8 minutes at 72 °C. Differences in the amplified alleles sizes and the use of different fluorescent dye labels of the primers enabled the pooling of multiple loci for the subsequent sequencing process. The pooled products were diluted with water at a ratio of 1:30, mixed with HiDiformamid and the internal size standard ROX500 (Applied Biosystems), and run on an ABI 3130xl Genetic Analyser. The program Peakscanner Software (Applied Biosystems) was used to inspect the alleles. Final allele sizes were determined using TANDEM v1.08 [2]. The program KINGROUP v2 [3] was used to determine pairwise relatedness coefficients *r* [4] for all possible dyads. The implemented simulation function was used to obtain reference intervals (first to third quartile) for expected pairwise relatedness values for 100 full siblings, 100 half siblings, and 100 unrelated individuals based on the actual allele frequencies of our focal population. Through this approach we obtained the most probable reference intervals for first- and second-order relatives, as well as for unrelated individuals, which were [0.365;0.573],[0.149;0.370], and [-0.120;0.122], respectively. Accordingly, we defined full-siblings as all individual pairs with *r*-

values > 0.368, half siblings with values between 0.135 and 0.368, and unrelated individuals with values below 0.135.

Sound analysis

See text file labelled "Praat script used to analyze defensive calls in Common ravens"

Sound manipulation

To shorten and lengthen the calls, the 'lengthen (overlap-add)' command was used (settings: time step=0.01s, minimum *f*o =75Hz, maximum *f*o =700Hz) and entered 1.5 as factor to lengthen the calls by 50%, or 0.5 to shorten the calls by 50 %. For *f*o manipulations, the *f*o contour was extracted from the original calls (see above) and time-stamped *f*o contours were created ('extract visible pitch contour' and 'down to PitchTier' commands). The frequency of the 'PitchTier' object was then shifted up and down by 100Hz ('modify' and 'shift frequencies' commands). The original *f*o was then replaced by the manipulated *f*o by creating a manipulation file from the original call ('To manipulation' command; settings: time step=0.01s, minimum *f*o =75Hz, maximum *f*o =700Hz), selecting both the newly created 'PitchTier' object and the manipulation file, and using the 'Replace pitch tier' command. The frequency manipulated sound files were resynthesized ('Get resynthesis (overlap-add)' command) and saved as wav-files.

Table S1: Model selection for all models investigating calling occurrences and number of calls per interaction bout. Corrected Akaike Information Criterion (AICc) values, the difference between the lowest AICc value and all other AICc values (Δi), the relative likelihood, and resulting Akaike weights are presented for each model. Bold type indicates models with highest support (Δi≦2). ":" denote interactions between factors.

**Model**	**AICc**	**Δi**	**Relative likelihood**	**Akaike weight**
Calling propensity				
**Level of aggression**	**960.38**	**0.00**	**1.000**	**0.500**
**Level of aggression + rank difference**	**961.24**	**0.86**	**0.650**	**0.325**
Level of aggression + kinship	964.35	3.97	0.137	0.069
Level of aggression + rank difference + kinship	965.19	4.81	0.090	0.045
Level of aggression*rank difference	966.37	5.99	0.050	0.025
Level of aggression*kinship	966.97	6.59	0.037	0.019
Level of aggression + rank difference + kinship + level of aggression:kinship	967.79	7.41	0.025	0.012
Level of aggression + rank difference + kinship + level of aggression:rank difference	970.32	9.94	0.007	0.003
Level of aggression + rank difference + kinship + level of aggression:rank difference + level of aggression:kinship (Full model)	972.87	12.49	0.002	0.001
Rank difference	1142.89	182.50	0.000	0.000
Null	1144.02	183.64	0.000	0.000
Rank difference + kinship	1145.53	185.15	0.000	0.000
Kinship	1146.90	186.51	0.000	0.000
Rank difference*kinship	1149.17	188.79	0.000	0.000
Number of calls/bout				
**Level of aggression**	**528.01**	**0.00**	**1.000**	**0.489**
**Level of aggression + rank difference**	**530.05**	**2.04**	**0.361**	**0.176**
Level of aggression + kinship	530.22	2.21	0.332	0.162
Level of aggression + rank difference + kinship	532.13	4.12	0.128	0.062
Level of aggression*kinship	532.59	4.58	0.101	0.049
Level of aggression*rank difference	533.73	5.72	0.057	0.028
Level of aggression + rank difference + kinship + level of aggression:kinship	534.47	6.46	0.039	0.019
Level of aggression + rank difference + kinship + level of aggression:rank difference	535.31	7.30	0.026	0.013
Level of aggression + rank difference + kinship + level of aggression:rank difference + level of aggression:kinship (Full model)	539.53	11.52	0.003	0.002
Null	552.36	24.35	0.000	0.000
Kinship	553.52	25.51	0.000	0.000
Rank difference	553.67	25.66	0.000	0.000
Rank difference + kinship	554.96	26.96	0.000	0.000
Rank difference*kinship	559.17	31.16	0.000	0.000

Table S2: Component matrix with loadings of the PCA including call duration (KMO=0.712, N=377).

Acoustic Variable	Principal Components			
	1	2	3	4
Minimum fo (Hz)	**0.97**	0.08	-0.01	0.00
Mean fo (Hz)	**0.97**	0.17	0.08	0.08
Maximum fo (Hz)	**0.94**	0.19	0.07	0.10
Start fo (Hz)	**0.93**	0.08	-0.02	-0.03
End fo (Hz)	**0.93**	0.09	-0.01	0.08
Mean amplitude (dB)	0.17	**0.97**	0.14	0.08
Minimum amplitude (dB)	0.10	**0.96**	0.17	-0.08
Maximum amplitude (dB)	0.19	**0.96**	0.13	0.12
Jitter	-0.15	-0.08	**-0.90**	-0.10
Tonality	-0.13	0.30	**0.83**	-0.12
Call duration	0.09	0.07	0.00	**0.99**
% of variance explained	47.24	25.08	11.16	9.13
Eigenvalue	5.20	2.76	1.23	1.00

Table S3: Model selection for all models investigating the three PCs and call duration. Corrected Akaike Information Criterion (AICc) values, the difference between the lowest AICc value and all other AICc values (Δi), relative likelihood, and Akaike weights are presented. Models with highest support (Δi≦2) are in bold type, and ":" denote interactions between factors.

**Model**	**AICc**	**Δi**	**Relative likelihood**	**Akaike weight**
PC1				
**Level of aggression*rank difference**	**823.09**	**0.00**	**1.000**	**0.777**
Level of aggression + rank difference + kinship + level of aggression:rank difference	826.51	3.42	0.181	0.141
Level of aggression	829.40	6.31	0.043	0.033
Level of aggression + rank difference	829.76	6.67	0.036	0.028
Level of aggression + rank difference + kinship	832.48	9.39	0.009	0.007
Level of aggression + kinship	832.54	9.44	0.009	0.007
Null	834.37	11.27	0.004	0.003
Rank difference	834.62	11.53	0.003	0.002
Level of aggression + rank difference + kinship + level of aggression:rank difference + level of aggression:kinship (Full model)	835.68	12.58	0.002	0.001
Kinship	838.31	15.21	0.000	0.000
Rank difference + kinship	838.35	15.26	0.000	0.000
Level of aggression*kinship	840.40	17.31	0.000	0.000
Level of aggression + rank difference + kinship + level of aggression:kinship	840.65	17.56	0.000	0.000
Rank difference*kinship	840.72	17.63	0.000	0.000
PC2				
**Level of aggression**	**635.61**	**0.00**	**1.000**	**0.582**
Level of aggression + rank difference	637.66	2.05	0.359	0.209
Level of aggression + kinship	639.64	4.02	0.134	0.078
Level of aggression*rank difference	639.91	4.30	0.117	0.068
Level of aggression + rank difference + kinship	641.69	6.08	0.048	0.028
Level of aggression*kinship	643.09	7.47	0.024	0.014
Level of aggression + rank difference + kinship + level of aggression:rank difference	643.86	8.25	0.016	0.009
Level of aggression + rank difference + kinship + level of aggression:kinship	645.23	9.62	0.008	0.005
Null	645.70	10.09	0.006	0.004
Rank difference	647.59	11.98	0.003	0.001
Kinship	647.71	12.09	0.002	0.001
Rank difference + kinship	649.76	14.15	0.001	0.000
Level of aggression + rank difference + kinship + level of aggression:rank difference + level of aggression:kinship (Full model)	650.10	14.49	0.001	0.000
Rank difference*kinship	650.32	14.71	0.001	0.000

PC3				
**Rank difference**	**987.47**	**0.00**	**1.000**	**0.724**
Rank difference + kinship	990.31	2.85	0.241	0.174
Level of aggression + rank difference	993.41	5.95	0.051	0.037
Rank difference*kinship	994.32	6.85	0.033	0.024
Null	994.47	7.00	0.030	0.022
Level of aggression + rank difference + kinship	996.60	9.14	0.010	0.008
Level of aggression*rank difference	997.11	9.64	0.008	0.006
Kinship	998.19	10.72	0.005	0.003
Level of aggression + rank difference + kinship + level of aggression:rank difference	1000.50	13.04	0.001	0.001
Level of aggression	1000.61	13.15	0.001	0.001
Level of aggression + kinship	1004.28	16.81	0.000	0.000
Level of aggression + rank difference + kinship + level of aggression:kinship	1006.34	18.87	0.000	0.000
Level of aggression + rank difference + kinship + level of aggression:rank difference + level of aggression:kinship (Full model)	1009.84	22.37	0.000	0.000
Level of aggression*kinship	1014.29	26.82	0.000	0.000
Call duration				
**Level of aggression*rank difference**	**-1310.43**	**0.00**	**1.000**	**0.874**
Level of aggression + rank difference + kinship + level of aggression:rank difference	-1306.28	4.15	0.126	0.110
Level of aggression + rank difference + kinship + level of aggression:rank difference + level of aggression:kinship (Full model)	-1302.42	8.00	0.018	0.016
Level of aggression	-1293.39	17.04	0.000	0.000
Level of aggression + rank difference	-1291.68	18.75	0.000	0.000
Null	-1291.35	19.08	0.000	0.000
Kinship	-1290.28	20.15	0.000	0.000
Rank difference	-1290.16	20.27	0.000	0.000
Level of aggression + kinship	-1289.59	20.84	0.000	0.000
Rank difference + kinship	-1288.52	21.91	0.000	0.000
Level of aggression*kinship	-1287.89	22.54	0.000	0.000
Level of aggression + rank difference + kinship	-1287.76	22.67	0.000	0.000
Rank difference*kinship	-1286.05	24.38	0.000	0.000
Level of aggression + rank difference + kinship + level of aggression:kinship	-1285.73	24.70	0.000	0.000

Table S4: Model selection for all models investigating the responses to playbacks of defensive calls manipulated in *f*o and call duration. Quasi AICc values (QAICc), the difference between the lowest QAICc value and all other QAICc values (Δi), relative likelihood, and quasi Akaike weights are presented. Models with highest support (Δi≦2) are in bold type.

Model	QAICc	Δi	Relative likelihood	QAkaike weight
Pitch manipulation				
**Manipulation type**	**37.1**	**0.0**	**1.000**	**0.606**
Manipulation type + sex of stimuli	39.6	2.6	0.275	0.166
Manipulation type + number of defensive calls	40.4	3.4	0.185	0.112
Null	42.6	5.6	0.062	0.037
Manipulation type + number of defensive calls + sex of stimuli	42.7	5.6	0.060	0.037
Number of defensive calls	44.3	7.2	0.027	0.016
Sex of stimuli	44.7	7.6	0.022	0.013
Number of defensive calls + sex of stimuli	45.0	8.0	0.018	0.011
Duration manipulation				
**Null**	**60.2**	**0.0**	**1.000**	**0.717**
Sex of stimuli	63.2	3.0	0.223	0.160
Number of defensive calls + sex of stimuli	65.7	5.5	0.064	0.046
Manipulation type + sex of stimuli	66.6	6.4	0.041	0.029
Number of defensive calls	66.6	6.5	0.039	0.028
Manipulation type	68.7	8.5	0.014	0.010
Manipulation type + number of defensive calls + sex of stimuli	69.8	9.6	0.008	0.006
Manipulation type + number of defensive calls	70.2	10.1	0.007	0.005

Table S5: Summary table of the number of agonistic interactions in the absence or presence of defensive calls with respect to the age class (A=adult, S=subadult, J=juvenile) and sex (F=female, M=male) of the opponents (the first letter indicates the sex or age class of the aggressor, and the second letter that of the victim). Numbers in brackets denote the number of birds per age class and sex.

Age class and sex of		Calling					No calling					Total
aggressor	victim	fight	forced retreat	retreat	sub-mission	Total	fight	forced retreat	retreat	sub-mission	Total	
AF (19)	AF	1	7	11	1	20	3	18	2	2	25	45
	AM	0	5	5	0	10	0	1	0	0	1	11
	SF	0	8	8	4	20	0	26	2	1	29	49
	SM	0	2	3	1	6	0	9	0	0	9	15
	JF	0	2	2	1	5	1	17	0	6	24	29
	FM	0	0	0	0	0	0	10	0	2	12	12
AM (22)	AF	1	28	22	1	52	3	39	1	0	43	95
	AM	6	23	33	1	63	3	30	5	2	40	103
	SF	0	22	23	3	48	0	24	0	6	30	78
	SM	0	20	27	9	56	0	35	8	3	46	102
	JF	0	6	7	0	13	3	34	0	0	37	50
	JM	0	2	4	0	6	0	22	0	4	26	32
SF (16)	AF	0	2	2	0	4	1	1	1	0	3	7
	AM	0	2	1	0	3	0	0	0	0	0	3
	SF	0	5	6	0	11	0	11	2	2	15	26
	SM	0	3	3	0	6	0	2	0	0	2	8
	JF	0	3	1	0	4	2	11	1	3	17	21
	JM	0	0	0	0	0	0	0	0	0	0	0
SM (15)	AF	0	18	3	0	21	5	9	1	0	15	36
	AM	0	1	1	0	2	1	2	0	0	3	5
	SF	0	19	17	0	36	0	16	1	1	18	54
	SM	0	9	8	0	17	3	15	4	1	23	40
	JF	0	1	0	1	2	0	13	0	0	13	15
	JM	0	1	0	0	1	0	8	0	0	8	9
JF (5)	AF	0	0	0	0	0	0	0	0	0	0	0
	AM	0	0	0	0	0	0	0	0	0	0	0
	SF	0	0	0	0	0	0	0	0	0	0	0
	SM	0	0	0	0	0	0	0	0	0	0	0
	JF	0	1	3	0	4	0	5	0	0	5	9
	JM	0	1	0	0	1	0	1	0	0	1	2
JM (3)	AF	0	0	0	0	0	0	0	0	0	0	0
	AM	0	0	0	0	0	0	0	0	0	0	0
	SF	0	1	2	0	3	0	2	0	0	2	5
	SM	0	0	0	0	0	0	0	0	0	0	0
	JF	0	0	2	0	2	0	2	0	0	2	4
	JM	0	0	0	0	0	0	0	0	0	0	0
Total		**8**	**192**	**194**	**22**	**416**	**25**	**363**	**28**	**33**	**449**	**865**

Table S6: Average acoustic measures and standard deviation for all acoustic parameters measured from the defensive calls.

Acoustic parameter	Level of aggression							
	fight		forced retreat		retreat		submission	
	mean	SD	mean	SD	mean	SD	mean	SD
Duration (s)	0.16	0.04	0.13	0.05	0.14	0.07	0.13	0.04
Mean *f*o (Hz)	432.15	65.97	410.23	55.36	382.45	51.64	329.93	105.16
Min *f*o (Hz)	361.54	59.09	346.14	58.89	316.91	52.66	276.76	88.53
T min *f*o (%)	55.41	39.45	58.33	33.48	54.90	39.26	64.54	36.27
Max *f*o (Hz)	464.87	70.45	442.75	57.44	413.42	58.41	356.14	114.32
T max *f*o (%)	44.13	14.13	45.35	18.15	46.88	15.17	43.67	14.12
*f*o range (Hz)	103.33	45.36	96.62	41.52	96.51	41.12	79.38	54.25
Start *f*o (Hz)	398.83	66.78	394.64	66.59	351.37	58.72	310.98	101.97
End *f*o (Hz)	380.36	66.63	368.73	71.66	337.26	59.60	289.34	98.97
Sum of *f*o variation	1254.13	596.41	1127.42	519.03	957.00	426.63	950.16	606.97
Mean AMP (dB)	68.65	9.50	67.51	7.34	65.12	7.39	58.71	7.51
Min AMP (dB)	64.35	10.67	62.98	9.17	61.96	8.45	56.57	7.61
T min AMP (%)	57.91	25.05	64.41	17.77	60.58	19.51	55.63	20.00
Max AMP (dB)	70.43	9.44	69.38	7.10	66.62	7.33	60.04	7.59
T max AMP (%)	47.91	15.81	45.55	13.82	45.69	13.44	48.05	13.97
AMP variation over time (dB/s)	57.14	27.79	68.28	63.20	52.08	47.54	44.73	38.56
Jitter	0.05	0.01	0.04	0.02	0.04	0.01	0.05	0.02
Harmonicity (dB)	5.38	1.69	5.72	1.72	5.84	1.74	4.75	1.39
Tonality (%)	74.85	18.49	73.59	25.73	68.07	22.57	69.83	27.45
Inflex (*f*o changes/s)	13.16	8.25	14.73	9.00	15.11	8.41	15.96	8.95

Note: AMP = amplitude; min = minimum, max = maximum, T = relative time

Supplementary References

1. Sambrook J, Fritsch EF, Maniatis T. Molecular cloning: A laboratory manual. 2nd ed. New York: Cold Spring Harbor Laboratory Press; 1989.

2. Matschiner M, Salzburger W. TANDEM: integrating automated allele binning into genetics and genomics workflows. Bioinformatics. 2009;25:1982–3.

3. Konovalov DA, Manning C, Henshaw MT. KINGROUP: a program for pedigree relationship reconstruction and kin group assignments using genetic markers. Mol Ecol Notes. 2004;4:779–82.

4. Queller DC, Goodnight KF. Estimating Relatedness Using Genetic Markers. Evolution. 1989;43:258–75.
